# Increasing Life Expectancy with Plant Polyphenols: Lessons from the Mediterranean and Japanese Diets

**DOI:** 10.3390/molecules30132888

**Published:** 2025-07-07

**Authors:** Marco Fiore, Anton B. Tonchev, Ruzha Z. Pancheva, Tetsumori Yamashima, Sabrina Venditti, Giampiero Ferraguti, Sergio Terracina

**Affiliations:** 1 Institute of Biochemistry and Cell Biology (IBBC-CNR), Department of Sensory Organs, Sapienza University of Rome, 00185 Rome, Italy; 2 Department of Anatomy and Cell Biology, Faculty of Medicine, Medical University, 9000 Varna, Bulgaria; anton.tonchev@mu-varna.bg; 3 NutriLect Study Group, Project No. BG-RRP-2.004-0009-C02, Medical University, 9000 Varna, Bulgaria; 4 Group on Early Childhood Intervention, Department of Hygiene and Epidemiology, Research Institute, Faculty of Public Health, Medical University, 9002 Varna, Bulgaria; 5 Department of Psychiatry and Behavioral Sciences, Kanazawa University Graduate School of Medical Science, Kanazawa 920-8641, Japan; 6 Department of Biology and Biotechnologies “Charles Darwin”, Sapienza University of Rome, 00185 Rome, Italy; sabrina.venditti@uniroma1.it; 7 Department of Experimental Medicine, Sapienza University of Rome, 00185 Rome, Italy

**Keywords:** plant polyphenols, longevity, mediterranean diet, Japanese diet, antioxidants, aging, nutraceuticals, vegan diet, vegetarian diet

## Abstract

Plant polyphenols have emerged as potent bioactive molecules that can modulate key cellular pathways associated with aging and chronic disorders. The Mediterranean diet and the traditional Japanese style of life are rich in polyphenol-containing foods and beverages, and epidemiological evidence links these dietary patterns to increased longevity and reduced morbidity. This narrative review examines the chemical description of plant polyphenols, their mechanisms of action, including anti-inflammatory, antioxidant, and hormetic effects, and how supplementation or a diet rich in these compounds may provide further life extension. We discuss the major classes of polyphenols present in the Mediterranean dietary pattern (e.g., resveratrol and hydroxytyrosol) and in the Japanese diet (e.g., epigallocatechin gallate and soy isoflavones), comparing their biological behaviors and cooperative effects on metabolic, cardiovascular, and neurodegenerative conditions. We also examine a few preclinical and clinical studies that explain the beneficial impact of these chemicals on aging-associated biomarkers. Furthermore, both dietary habits are characterized by low consumption of processed foods and sugary carbonated drinks and reduced utilization of deep-frying with linoleic acid-rich oils, a practice that reduces the formation of harmful lipid peroxidation products, notably 4-hydroxynonenal, known to be implicated in accelerating the aging process. The Mediterranean dietary pattern is also characterized by a low/moderate daily consumption of wine, mainly red wine. This work debates emerging evidence addressing issues of bioavailability, dosage optimization, and formulation technologies for polyphenol supplementation, also comparing differences and similarities with the vegan and vegetarian diets. We also explore how these chemicals could modulate epigenetic modifications that affect gene expression patterns pertinent to health and aging. In conclusion, we aim to show a consolidated framework for the comprehension of how plant polyphenols could be utilized in nutritional strategies for potentiating life expectancy while stimulating further research on nutraceutical development.

## 1. Introduction

The contemporary search for increased life expectancy and enhanced quality of life has strengthened interest in nutritional interventions that could regulate aging processes at the molecular level. Aging is a multifactorial event influenced by genetic, environmental, and dietary factors [1,2,3]. A growing body of evidence suggests that chronic inflammation and oxidative stress elicit the decline of cellular function, finally leading to age-related disorders such as cardiovascular diseases, neurodegenerative conditions, and certain types of tumors [4,5,6,7,8,9,10]. Polyphenols—a diverse group of naturally occurring chemicals found in vegetables, fruits, wines, teas, and extra-virgin olive oil—show a compelling role in mitigating these deleterious processes [11,12,13,14,15].

The Mediterranean diet, characterized by an elevated intake of plant-based foods, nuts, extra-virgin olive oil, and moderate wine consumption, has recurrently been associated with enhanced cardiovascular health and increased lifespan [16,17,18,19,20,21,22]. Similarly, traditional Japanese dietary patterns, which accentuate soy products, vegetables, seaweed, and green tea, have been related to longevity and low occurrences of dementia and metabolic conditions [23,24,25,26,27,28,29]. Although the two dietary styles originate from divergent culinary and cultural traditions, a common feature is the profusion of polyphenolic chemicals that seem to confer protective effects.

In this narrative review, we discuss the hypothesis that supplementation with plant polyphenols, whether across selected dietary interventions or concentrated nutraceutical formulations, could positively impact life expectancy. We first aim to review the chemistry and biochemistry of polyphenols and describe their mechanistic functions, including free radical scavenging, the modulation of inflammatory paths, and the stimulation of cellular defense mechanisms. We then explore detailed analyses of the polyphenol-rich Mediterranean and Japanese diets to demonstrate how long-term adherence to these dietary patterns could correlate with reduced mortality rates. By integrating data from clinical trials, epidemiological studies, and preclinical research, we postulate an integrated assessment of the potential mechanisms underlying polyphenols’ longevity-promoting effects. We also aim to discuss future directions in this field, including approaches for potentiated bioavailability and innovation in supplementation procedures.

The objective of this review is twofold: to provide a comprehensive indication of the current scientific comprehension of plant polyphenols, their protein substrates that protect against oxidation injury, and their role in counteracting age-related decline, and to gain insights into how lessons from two of the world’s healthiest dietary lifestyles can be utilized to improve longevity. Indeed, a better comprehension of these mechanisms may incentivize the development of targeted nutritional approaches aimed at optimizing health extent and reducing the weight of chronic disorders linked to aging.

## 2. Materials and Methods

In April 2025, an accurate literature search was carried out to identify crucial studies across multiple databases, including Scopus, PubMed, and Web of Science, to perform this narrative review. Papers were recruited using keywords such as “health”, “polyphenols”, “Mediterranean diet”, “Japanese diet”, “inflammation”, “neurodegeneration”, “cardiovascular conditions”, “metabolic disorders”, and “oxidative stress” without a limit on publication year. Delimited inclusion criteria were as follows: (1) English-language articles, (2) original studies on the above-mentioned keywords, and among polyphenols we considered in the discussion resveratrol, epigallocatechin gallate (EGCG), quercetin, hydroxytyrosol/oleuropein/tyrosol (olive polyphenols). Letters, editorials, and case reports were not included. Studies following these criteria were further evaluated, and pertinent findings were extracted from each work.

## 3. Plant Polyphenols: Chemistry and Mechanisms of Action

### 3.1. Classification and Chemical Description

Plant polyphenols are a vast family of secondary metabolites and are generally divided according to their chemical structure into numerous major classes [30,31,32]:

- Flavonoids [33,34]: This group includes flavanols (e.g., EGCG), flavones (e.g., luteolin), flavonols (e.g., quercetin and kaempferol), flavanones, neoflavonoids, isoflavones (e.g., Genistein and Daidzein), and anthocyanins.
- Phenolic acids [35]: Such as ferulic acid and caffeic acid.
- Stilbenoid [36,37]: With resveratrol being one of the most famous.
- Lignans [38]: Discovered in whole grains and numerous seeds.
- Tannins [39,40]: A class of astringent, polyphenolic biomolecules such as gallic acid.

These chemicals are characterized by one or several hydroxyl groups on aromatic (phenolic) rings and show noteworthy redox properties. Their ability to donate electrons permits them to be potent free radical scavengers, so rapidly acting against oxidative damage at the physiological level [11,41,42,43].

### 3.2. Antioxidant and Anti-Inflammatory Mechanisms

Plant polyphenols elicit their biological action mainly through three related mechanisms:

1. Reactive Oxygen Species (ROS) Direct Scavenging [44,45,46,47,48]: By counteracting free radicals such as hydroxyl radicals and superoxide anions, plant polyphenols may neutralize oxidative damage directly to DNA, lipids, and proteins.
2. Fine Regulation of Cellular Signaling: Many plant polyphenols modulate key intracellular pathways, involving the activation of the nuclear factor erythroid 2-related factor 2 (Nrf2), which has a crucial role in the expression of antioxidant peptides [49,50,51,52]. Furthermore, they reduce the nuclear factor kappa-light-chain-enhancer of the activated B cell (NF-κB) path, thus decreasing the activity of pro-inflammatory cytokines [11,53,54,55,56].
3. Hormetic Actions: Hormesis is a two-step dose–response association with an environmental agent, whereby low-dose amounts may have a positive effect and high-dose quantities could be functionally inhibitory or toxic [57,58]. Indeed, at low doses, polyphenols may elicit a minor stress response aimed at enhancing cellular resilience, which may modulate stress resistance and regulate endogenous repair mechanisms [59,60,61,62].

These processes represent the biochemical basis for the protective actions of polyphenols against the development of age-related conditions, supporting the hypothesis that regular polyphenol consumption might slow down aging progression.

### 3.3. Polyphenols’ Role in Cellular Metabolism and Longevity

At the biomolecular level, polyphenols also regulate pathways with important roles in mitochondrial biogenesis, autophagy, and apoptosis [63,64,65,66]. EGCG, a main catechin discovered in green tea, has been shown to modulate signaling pathways associated with inflammation and cell survival [67,68,69]. Other polyphenols impact the above pathways by means of epigenetic mechanisms. For example, resveratrol, a well-known stilbene, can modulate the actions of sirtuins [70,71,72], a class of chromatin-modifying enzymes that control cellular responses to stress and have been associated with potentiated lifespan in numerous organisms [73,74,75].

**Figure 1:**
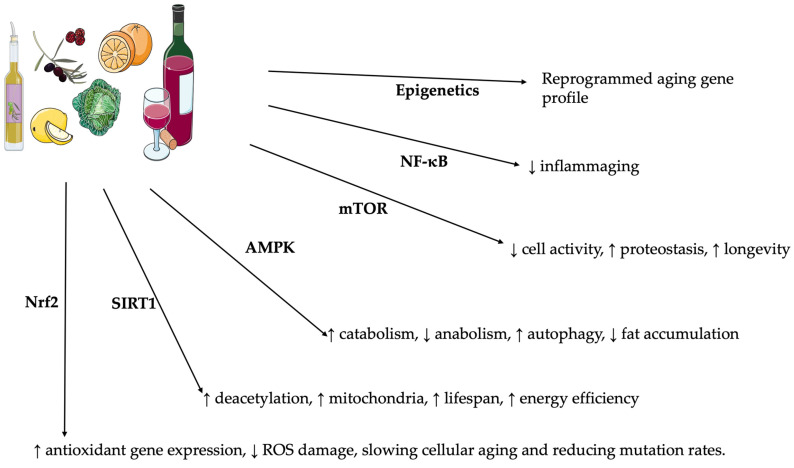
Polyphenol pathways and aging. NF-kB (nuclear factor kappa-light-chain-enhancer of activated B cells); mTOR (mammalian target of rapamycin); AMPK (5′ AMP-activated protein kinase); SIRT1 (sirtuin 1); Nrf2 (nuclear factor erythroid 2-related factor 2). Servier Medical Art by Servier is licensed under a Creative Commons Attribution 3.0 Unported License (https://creativecommons.org/licenses/by/3.0/, accessed on 29 June 2025). ↑ indicates elevation. ↓ indicates reduction.

Moreover, polyphenols may induce epigenetic modifications with a synergistic action with microRNAs [76,77,78,79], thus regulating gene expression patterns related to aging. This multifaceted approach allows polyphenols to subtly remodel cellular homeostasis and participate in a healthier and longer lifespan (Figure 1).

## 4. The Mediterranean Diet: A Polyphenol-Rich Nutritional Paradigm

### 4.1. Dietary Pattern and Polyphenol Sources

The Mediterranean diet is renowned for its emphasis on vegetables, fresh fruits, whole grains, legumes, nuts, and, particularly, extra-virgin olive oil [3,17,80,81,82]. Moderate consumption of wine (mainly red wine)—rich in resveratrol and other polyphenols—is another distinct characteristic [15,83,84,85,86,87,88,89,90]. Indeed, a wide variety of plant foods deliver an extensive selection of polyphenols, each with distinctive biological activities and chemical properties.

Key polyphenolic compounds found in the Mediterranean diet include the following:

- Hydroxytyrosol, Tyrosol, and Oleuropein: Mostly found in olive leaves and extra-virgin olive oil, these chemicals display robust antioxidant and anti-inflammatory capabilities [85,91,92,93,94].
- Resveratrol: Found in the skin of grapes, blueberries, raspberries, mulberries, and peanuts [95,96,97], resveratrol has been associated with the activation of longevity-related pathways such as the Phosphoinositide 3-Kinase/Protein Kinase B (PI3K/Akt), Sirtuin 1 (SIRT1), and AMP-Activated Protein Kinase (AMPK) pathways [98,99,100,101] while inhibiting MTOR (mammalian target of rapamycin), a protein kinase that plays a crucial role in cell growth, proliferation, and metabolism [102].
- Flavonoids: These are abundant in vegetables and fruits (e.g., quercetin in radish leaves, radicchio, tomatoes, and red onions; catechins in tea) that participate in the total antioxidative shape of the diet [103,104,105,106,107].

### 4.2. Health Outcomes Correlated with the Mediterranean Diet

Several epidemiological studies have associated the Mediterranean diet with lower percentages of certain tumors, decreased incidences of metabolic syndrome, and enhanced cardiovascular conditions [108,109,110,111,112]. For example, the PREDIMED (Prevención con Dieta Mediterránea) trial showed convincing evidence that a Mediterranean dietary pattern supplemented with encouraged low-fat food items, nuts, or extra-virgin olive oil lowers the risk of the main cardiovascular disorders [113,114]. These findings, combined with observed improved lipid profiles and reductions in inflammatory markers, strongly imply that a diet enriched in high plant polyphenol content may have a key role in potentiating protective actions [115].

The interaction between different polyphenols emerges as particularly significant. When assumed as part of a whole diet, these chemicals may have additive or even accumulating effects on health, due to their capability to concurrently target numerous signaling cascades. This multifaceted interplay of dietary elements configures the Mediterranean dietary pattern as a nutritional style that effectively counteracts inflammation and oxidative stress, both of which are key factors in age-associated diseases.

### 4.3. Further Elements from Mediterranean Diet Studies

Other biomolecular studies have shown that polyphenol-enriched extracts from Mediterranean foods can potentiate *(i)* the production of neurotrophins (namely brain-derived neurotrophic factor, BDNF, and nerve growth factor, NGF) [116,117,118] and *(ii)* endothelial function [119,120,121,122,123], and *(iii)* stimulate nitric oxide (NO) release—a crucial cause in preserving vascular health [124,125,126,127].

Moreover, these compounds support the cell transition from a pro-inflammatory up to an anti-inflammatory condition by regulating cytokine shapes, suppressing the expression of genes involved in pro-inflammatory molecules’ release, such as toll-like receptor (TLR), and decreasing the levels of C-reactive protein (CRP) [11,128,129,130]. It should be noted that several investigations have correlated such biomolecular improvements with decreased arterial rigidity and elevated overall cardiovascular functioning, which may in turn contribute to longer life expectancy [131,132].

By diminishing oxidative injury and reducing inflammatory responses at a systemic level, the observance of the Mediterranean dietary pattern could offer a molecular environment leading to better healthy aging. Elucidating the substrate of polyphenols and its protection would be crucial for both decreasing cell degeneration/death and increasing life expectancy. This dietary practice could not only delay the beginning of age-related conditions but also increase the life quality in the elderly—a dichotomy that emphasizes its potentiality as a prototype for nutritional interventions designed to extend the lifespan.

## 5. Japanese Dietary Patterns and the Role of Polyphenols in Longevity

### 5.1. Traditional Japanese Diet and Its Polyphenol Profile

Japan is recognized for having one of the world’s highest life expectancies [133]. This fact could be somewhat attributable to the traditional Japanese dietary practice, which is certainly elevated in marine/soy-based proteins, low in saturated fats, and rich in vegetables and beverages containing high amounts of polyphenols, mostly green tea [25,28,134]. Green tea, which is high in EGCG, represents a major contributor to the dietary polyphenol burden in Japan. Other important components encompass soy-based elements (e.g., miso and tofu, containing genistein and daidzein) that offer isoflavones and a wide plethora of sea vegetables that provide distinctive phenolic chemicals [135,136].

### 5.2. Epidemiological Proof of Longevity

The intake of green tea has been recurrently associated with decreased incidences of certain tumors, cardiovascular disorders, and neurodegenerative conditions. Epidemiological studies in Japan display that regular green tea consumers show lower mortality rates compared to non-drinkers—an advantage attributed not only to EGCG but also to the general phytochemical setting of Japanese dietary practice [137,138,139]. Isoflavones from soy, for instance, might contribute to enhanced bone density and decreased risk of hormone-dependent tumors, while seaweed-derived molecules have been related to lower blood cholesterol presence and potentiated thyroid function [140,141,142,143].

Several cohort investigations have clearly shown relationships between elevated urinary polyphenol concentrations, mirroring a dietary style abundant in these chemicals, and positive health outcomes. These findings suggest that the growing effects of daily assumption of modest amounts of different polyphenols can cause a biologically valuable “polyphenol signature” that could decelerate aging events and potentiate cellular defense mechanisms [144,145,146].

### 5.3. Biomolecular Mechanisms of the Japanese Dietary Schedule

Investigations on the biomolecular actions of green tea polyphenols have clarified some pathways pertinent to longevity. Indeed, EGCG has been demonstrated to facilitate mitochondrial function, activate critical metabolic controllers (e.g., AMPK), and decrease cellular aging [147,148,149,150]. EGCG also activates the Keap1–Nrf2/ARE axis and up-regulating phase II antioxidant enzymes and glutathione synthesis, restoring redox homeostasis in neurons and hepatocytes [147,148,149,150,151,152,153]. Furthermore, EGCG blocks NF-κB nuclear translocation, reducing pro-inflammatory cytokine (e.g., TNF-α and IL-6) production in the vascular endothelium and microglia [154,155,156,157,158,159].

The soy isoflavones (i.e., genistein and daidzein) of the Japanese dietary patterns possess slight estrogen-like properties that support the maintenance of skeletal and cardiovascular health, mostly in postmenopausal women [151,152,153]. Furthermore, soy isoflavones might *(i)* modulate PI3K/Akt/eNOS (phosphatidylinositol 3-kinase/protein kinase B/endothelial nitric oxide synthase) signaling in endothelial cells, eliciting vasodilation and inhibiting vascular smooth muscle cell proliferation [160,161]; *(ii)* inhibit NADPH (nicotinamide adenine dinucleotide phosphate) oxidase isoforms and down-regulate reactive oxygen species in cardiomyocytes [162]; and *(iii)* stimulate epigenetic actions by regulating DNA methyltransferase, histone deacetylase, and microRNA expression (e.g., miR-155 and miR-21) known to contribute to anti-inflammatory and anti-fibrotic gene expression profiles [163,164,165,166].

In addition to these straight outcomes, the traditional lifestyle of Japanese people—embracing communal eating patterns and mild physical activity, and placing a cultural importance on balance—underlines the beneficial influence of dietary polyphenols by decreasing stress and endorsing general well-being [167,168]. This multifactorial method not only reduces the inflammatory load but also elicits a complex metabolic setting that supports longevity.

## 6. Relationship Between Mediterranean and Japanese Polyphenol Contents

### 6.1. Major Polyphenolic Presence Characterizing the Japanese/Mediterranean Diet Profiles

A qualitative evaluation of the two dietary schedules reveals different but complementary polyphenol paradigms. Table 1 highlights some of the key polyphenolic chemicals prevalent in each dietary pattern.

**Table 1:** Major polyphenol elements found in the Mediterranean and Japanese diets. This table shows that while each dietary paradigm provides a distinct composition, the essential similarity in the types of polyphenols—such as flavonoids and catechins—indicates a convergent evolution dealing with nutrition that boosts cellular health and antioxidant defenses.

Polyphenolic Class	Mediterranean Diet	Japanese Diet
Stilbenes	Resveratrol (from red wine, grapes, and berries) [169]	–
Phenolic Alcohols	Hydroxytyrosol, tyrosol, and oleuropein (from extra-virgin olive oil and olive leaves) [81]	–
Flavonoids	Quercetin and catechins (from fruits/vegetables) [170]	Catechins (EGCG from green tea) [171]
Isoflavones	–	Genistein and daidzein (from soy products) [172]
Other Phenolic Chemicals	Various minor polyphenols (nuts and legumes) [173]	Unique compounds from seaweeds and mushrooms [174]

### 6.2. Cooperative Effects and Nutrient Connections

**Figure 2:**
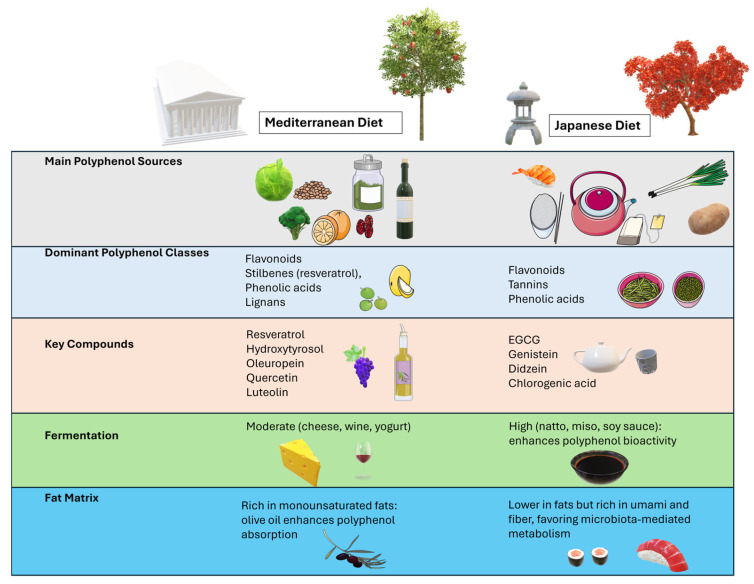
Relationship between Mediterranean and Japanese polyphenol contents. The Mediterranean and Japanese diets are both associated with exceptional health benefits and longevity, and a key shared feature is their high content of polyphenols. However, these diets differ in polyphenol sources, types, and patterns of consumption, leading to distinct metabolic profiles and potentially complementary effects on aging and disease prevention. EGCG, epigallocatechin gallate. Parts of the figure were drawn by using pictures from Servier Medical Art and Microsoft PowerPoint 365 Version 2112 (https://www.microsoft.com/microsoft-365, accessed on 29 June 2025). Servier Medical Art by Servier is licensed under a Creative Commons Attribution 3.0 Unported License (https://creativecommons.org/licenses/by/3.0/, accessed on 29 June 2025).

The holistic advantages derived from the Mediterranean and Japanese diets are rarely caused by a single chemical in isolation. Instead, their effectiveness seems to be established by combinatory interplay among several nutrients. For instance, the consumption of extra-virgin olive oil in the Mediterranean diet not only provides hydroxytyrosol/tyrosol/oleuropein but also supplies monounsaturated fats that contribute to effective energy metabolism and cell membrane integrity [175,176,177,178]. Likewise, the Japanese diet’s emphasis on soy products and green tea originates from a biochemical environment in which the distinct actions of isoflavones and EGCG strengthen each other to regulate pathways dealing with stress-responsive signaling [135,179] (Figure 2).

Previous investigations in nutritional biochemistry have shown that the collective antioxidant ability of a mixed polyphenol-enriched diet may be significantly superior to the sum of its components [154,155,156]. These data strongly sustain the hypothesis that a wide dietary paradigm, rather than single-compound supplementation, could be the ideal strategy for exploiting the full range of polyphenol benefits.

### 6.3. The Impact on Aging Critical Biomarkers

Both diets have been correlated with encouraging modifications in biomarkers associated with inflammatory conditions and oxidative stress. Individuals strictly adherent to the Mediterranean diet often display a lower plasma presence of oxidized low-density lipoprotein (ox-LDL), decreased inflammatory cytokine concentrations, and better endothelial functionality [157,158,159]. Comparably, regular drinkers of green tea in Japan show a lower presence of pro-inflammatory biomarkers, potentiated mitochondrial efficacy, and elevation in protective genes associated with longevity [180,181,182].

In both circumstances, the long-term consumption of polyphenol-rich foods seems to participate in reduced cellular senescence and enhanced resilience against metabolic problems. Such molecular biomarkers are progressively being documented in the scientific literature as prognosticators of increased quality of life and life expectancy in the elderly.

## 7. Preclinical and Clinical Findings

### 7.1. Preclinical Studies

Animal models [183,184,185,186,187,188] and in vitro studies [189,190,191,192,193,194] have shown significant information on the mechanisms by which polyphenol supplementation may potentiate life expectancy. In rodent studies, resveratrol administration improved mitochondrial function and induced a retardation at the beginning of age-related decline [195,196,197,198,199]. Likewise, supplementation of EGCG in different studies has revealed reductions in biomarkers of oxidative stress [200,201,202,203] and augmented neuronal survival in animal models of neurodegeneration [204,205,206,207,208].

Mouse studies also disclosed a role played by NGF and BDNF in modulating oxidative stress and brain functionality following olive polyphenol and/or resveratrol administration [82,116,117,118]. These data not only emphasize the critical role played by polyphenols as anti-aging components but also support the hypothesis describing how dietary patterns rich in these antioxidant chemicals could finely modulate cellular homeostasis.

### 7.2. Clinical Studies and Epidemiological Findings

Several human investigations have tried to translate these preclinical data into clinical evidence. For instance, randomized controlled trials (e.g., PREDIMED and CARDIOPREV) investigating the Mediterranean dietary pattern have constantly shown improvements in cardiovascular risk elements—such as arterial stiffness, circulating inflammatory biomarkers, and blood lipid presence—known to be crucial longevity determinants [22,209,210,211]. Observational studies carried out in Japan (e.g., Japan Public Health Center-based Prospective Study) have analogously discovered an inverse association between green tea drinking and all-cause morbidity and mortality [137,212,213].

Moreover, according to biomarker investigations, it has been shown that individuals with a greater baseline presence of blood polyphenols display a reduced incidence of neurodegenerative conditions and a decreased occurrence of metabolic syndrome [105,214,215,216]. Based on these considerations, the human findings indicate that plant polyphenol supplementation—whether via rigid dietary approaches or as supplemented nutraceuticals—can elicit significant effects on the biomolecular pathways that trigger aging and chronic conditions.

It should be noted, however, that unpredictability in polyphenol metabolism and bioavailability among individuals is a noteworthy challenge in nutrition. Clinical results are influenced by many factors, such as genetic polymorphisms regulating metabolic enzymes, the food substrate in which polyphenols are carried, and the gut microbiota configuration. Identifying these biases could be an essential tool for planning future studies and for the eventual personalization of polyphenol-based dietary patterns (Table 2).

## 8. The Impact of Sugary Carbonated Drinks, Processed Foods and Cooking Practices on Hydroxynonenal Formation on Aging Acceleration

An unacknowledged dietary issue contributing to aging is the presence of reactive aldehydes, particularly 4-hydroxynonenal (HNE) [229,230,231,232,233]. HNE originates during the deep-frying of linoleic acid-rich cooking oils; under high-temperature conditions, the unsaturated bonds in linoleic acid oxidize, leading to lipid peroxidation and HNE formation [229]. This toxic element can make adducts with nucleic acids, proteins, and other cellular constituents, thus eliciting oxidative stress, cellular abnormalities, neurodegeneration, and the progression of age-related conditions [229,230,231,232,233,234].

Intriguingly, after consecutive injections of synthetic HNE in monkeys in which the serum concentration reached that observed in humans in their 60s, significant brain, pancreas, and liver damage were observed. HNE elevation, combined with age-dependent ischemia in humans, may overactivate μ-calpain, which can cleave the lysosomal membrane stabilizer, Hsp70.1, especially after its specific oxidation injury, i.e., carbonylation by ROS. The ‘calpain–cathepsin hypothesis’ formulated by Yamashima et al. postulates calpain-mediated damage of lysosomal limiting membranes and subsequent cathepsin release [229,230,231,232,233]. This represents a central cascade for programmed cell death in diverse organs and leads to the occurrence of lifestyle-related diseases.

Another modern dietary concern is the extensive consumption of sugary carbonated or not beverages. These drinks are not only characterized by highly refined sugars but might also be highly processed. Indeed, regular consumption of drinks enriched in sugar can lead to severe pathological conditions such as obesity, type 2 diabetes mellitus, and cardiovascular disorders, and the formation of several glycation end products (AGEs) [235,236,237,238,239]. Indeed, AGEs, in turn, participate in the elevation of oxidative stress [240,241,242] and have been associated with the elevation of lipid peroxidation processes that may further increase HNE presence [243,244,245]. The metabolic abnormalities induced by high sugar intake, such as chronic inflammation and insulin resistance, can potentiate the harmful actions of HNE, increasing the risk of chronic degenerative disorders and accelerating cellular senescence.

A further modern dietary concern is the abuse of ultraprocessed and processed foods. They are characterized by their wide industrial modification, which dramatically modifies both their nutritional features and chemical structures compared to normal or marginally processed foods [246,247,248]. These edulis elements are generally overloaded with high amounts of refined sugars and unhealthy fats, mainly linoleic acid from specific seed oils, and a wide collection of artificial additives, emulsifiers, and preservatives. It should be noted that the industrial processes may consist of high-temperature cooking and chemical management, facilitating broad lipid peroxidation with the formation of HNE. Accordingly, the systematic consumption of ultraprocessed foods might compromise metabolic homeostasis, potentiating oxidative stress and stimulating unhealthy epigenetic modifications that further accelerate several degenerative processes associated with aging.

In this regard, both the Mediterranean diet and the Japanese style of life express a common culinary legacy: an inclination to consume fresh, minimally processed foods, limiting frequent deep-frying procedures. In these dietary paradigms, oil cooking techniques such as light sautéing, boiling, or steaming are preferred over deep-frying, which reduce the exposure to the high temperatures that stimulate linoleic acid oxidation. Furthermore, the selection of cooking oils further characterizes these dietary patterns. For example, extra-virgin olive oil, commonly utilized in Mediterranean cuisine, displays a higher oxidative solidity and an inferior linoleic acid presence compared to many industrial seed oils [249,250]. Likewise, traditional Japanese cuisine accentuates the utilization of fresh components and seasonal vegetables according to methods that do not produce great lipid peroxidation.

The take-home message of these practices is a decreased in vivo load of HNE and other damaging lipid peroxidation components. By limiting sugary carbonated drinks, processed foods, and deep-frying of linoleic acid-rich oils, these dietary practices might not only decrease the generation of toxic metabolites but also contribute to preserving cellular functions, reducing chronic inflammatory processes. Accordingly, the reduction in HNE accumulation may be a key point in the decreased incidence of age-related conditions enhancing longevity detected in individuals following these nutritional lifestyles.

## 9. The Dual Janus-Faced Role of Alcohol Consumption Within These Dietary Patterns

Alcohol drinking, in any form (e.g., wine, beer, and spirits), possesses a distinctive position in the context of dietary paradigms, including in the Mediterranean area due to early cultural motivations, exhibiting a dualistic nature that is extremely dependent on the type and quantity of intake. Indeed, in the Mediterranean diet, moderate consumption—mainly in the form of red wine—has been correlated with different cardiometabolic benefits [20,80,251,252]. However, chronic or excessive alcohol drinking is established to induce a wide spectrum of adverse health effects, from cancer to accelerated aging [253,254].

### 9.1. Toxic Effects of Alcohol Abuse

The abuse of alcohol is associated with several toxic outcomes [255,256]. At any dose, alcohol is metabolized into acetaldehyde, an extremely reactive chemical capable of triggering protein and DNA adducts [257,258,259,260,261,262,263]. This phenomenon not only contributes to liver disorders, such as alcoholic hepatitis, liver tumors, and cirrhosis, but also elevates the risk of cardiovascular diseases, certain types of cancer, and neurodegeneration. The chronic consumption of large amounts of alcohol affects cellular homeostasis, promoting systemic inflammatory processes and inducing cellular senescence—a plethora of effects that are well documented to decrease life expectancy, affecting the quality of life in the elderly.

### 9.2. Alcohol Drinking During Pregnancy: Fetal Alcohol Spectrum Disorders

Fetal alcohol spectrum disorders (FASDs) are a group of pathological conditions that can happen in an individual who was exposed to ethanol during pregnancy [264,265,266,267,268,269]. In Western countries, FASD affects between 1 and 4 in 20 newborns but is probably misdiagnosed and underdiagnosed [264,270]. The more severe form of FASD is fetal alcohol syndrome (FAS). The main mandatory signs for FAS diagnosis include growth deficiency or failure to grow, congenital malformation of the lips, short palpebral fissure lengths, and nervous system damage associated with functional impairments [271,272,273,274,275]. Quite surprisingly, in a mouse model of FASD carried out with red wine or alcohol per se supplementation, some of the deleterious effects of alcohol were counteracted when ethanol was administered as red wine, showing a sort of protection due to the natural presence of polyphenols in the wine [276,277,278,279]. Furthermore, resveratrol restored Nrf2 levels and prevented ethanol-induced toxic effects in the cerebellum of a rodent model of FASD [280], while curcumin did not lead to protective effects in growing zebrafish embryos [281]. It should also be noted that paternal alcohol exposure during mating may elicit outcomes in the offspring/newborns comparable to those observed for FASD [282,283,284].

### 9.3. Effects of Moderate Alcohol Consumption

Intriguingly, different investigations have shown that moderate consumption of alcohol—particularly wine in the Mediterranean area—seems to provide health benefits that could contribute to longevity [20,285,286]. Indeed, red wine is a natural source of polyphenolic chemicals such as resveratrol, anthocyanins, and quercetin [20,55,287], biomolecules known to induce antioxidant, cardioprotective, and anti-inflammatory effects. Epidemiological findings from Mediterranean countries, although debated, indicate that low-to-moderate red wine intake (circa one glass per day for non-gestating women and up to two for men) is associated with better endothelial function, a decrease in circulating inflammatory biomarkers, and potentiated lipid patterns [288,289,290,291]. In these conditions, the helpful actions of these polyphenols seem to counteract the potentially damaging metabolic effects of alcohol, assisting in the maintenance of a favorable oxidative and inflammatory equilibrium that could lead to healthy aging.

### 9.4. Balancing the Dual Effects: A Context-Dependent Paradigm

The “Janus face” of alcohol drinking emphasizes the crucial importance of the circumstance of its consumption. Within the frame of a dietary pattern characterized by low sugar intake and low ultraprocessed/processed food intake but abundant in protective natural polyphenols—as typified by the Mediterranean or Japanese diet—the moderate presence of good-quality wine could be integrated as a secondary element of a healthful lifestyle. Nevertheless, it is important to underscore that the health benefits identified in such populations rely upon a strict adherence to low/moderate consumption indications. According to the knowledge dealing with alcohol abuse, the toxic effects of consumption prevail, moving the balance toward inflammation, oxidative stress, and cellular injury, all of which contribute to accelerated aging and several disorders’ development.

## 10. The Close Relationship Between the Mediterranean/Japanese Diets and the Vegan/Vegetarian Diets

While the Mediterranean and Japanese diets both highlight polyphenol-rich whole foods, firm vegan and lacto-ovo vegetarian profiles also provide high total polyphenol content, often matching or exceeding those of other plant-related regimens, but with different characteristics [292,293,294,295,296,297,298,299,300]. For instance, a soy-centric vegetarian diet may provide more isoflavone content (genistein and daidzein) than the Japanese diet. Further, fruit-heavy vegan regimens could offer elevated levels of anthocyanins and flavonols.

A strict diet rich in nuts, whole grains, legumes, and a wide array of vegetables could strengthen both the vegan and vegetarian polyphenol assortment. However, the absence of wine stilbenes (resveratrol), extra-virgin olive oil polyphenols, and marine phlorotannins could limit their coverage of redox and anti-inflammatory properties.

The fat content further distinguishes these dietary patterns. Vegan and vegetarian diets are generally based on polyunsaturated-rich oils (e.g., walnut and flaxseed) and nut butters, which can modify micelle formation and the intestinal uptake of lipophilic polyphenols compared to the monounsaturated-rich extra virgin olive oil of the Mediterranean dietary pattern. Thus, different lipid transporters could modulate both the plasma content and tissue distribution of crucial antioxidant chemicals.

However, it should be noted that epidemiological data has established the health benefits among all four plant-based approaches. Several cohort investigations link vegan and vegetarian diets to lower all-cause mortality [292,293,294,295,296,297,298,299,300], including reduced incidence of cardiovascular disorders by improving the glycemic responses, findings comparable to those observed in Mediterranean and Japanese regimens. Thus, these parallel clinical outcomes, such as preserved bone density in soy consumers or the cardiometabolic advantages of Mediterranean dietary polyphenols, show that each regimen acts through sometimes non-overlapping arrangements of bioactive compounds.

It should also be considered that rigorous diets based only on vegetables could require subtle attention to micronutrients, particularly vitamin B12, iron, zinc, and iodine, chemicals known to play fine roles in the modulation of immune ability, mitochondrial function, and redox balance. Indeed, the unbalanced status of these co-factors could impair polyphenol action by disrupting antioxidant enzyme actions and eliciting low-grade inflammation.

## 11. Future Assessments and Proposals

### 11.1. Improving Polyphenol Administration

Investigations regarding the formulation of polyphenol supplements are promptly proceeding. Novel encapsulation methods, including liposomal carriage and nano-emulsion systems, are being established to better improve the bioavailability of these chemicals [301,302,303]. Indeed, many industrial plans have been developed to disclose effective dosages and combinations that optimize the biological effectiveness of polyphenols while minimizing metabolism-related outcomes.

The adoption of a unifying approach that blends whole-food dietary paradigms with scientifically established nutraceuticals could offer a double strategy for potentiating health span and life expectancy. Indeed, individuals incapable of strictly adhering to a Mediterranean or Japanese dietary pattern due to several reasons, including geographic, cultural, or socioeconomic explanations, might take advantage of directed supplementation formulas that resemble the polyphenol characterization of these diets.

### 11.2. Investigating Metabolic Contents and Bioavailability

The pharmacokinetic qualities of polyphenols change widely and features such as food processing, chemical organization, and interaction with other dietary elements severely influence polyphenols’ absorption and systemic spreading. Recent discoveries in gut microbiome research and metabolomics are beginning to show how these elements modulate the efficacy of polyphenols [304,305,306]. Forthcoming clinical studies should amalgamate these indications, utilizing the stratification of the microbiota composition and/or potential genetic markers to predict which individuals are expected to take advantage of targeted polyphenol supplementation.

### 11.3. Integration with Lifestyle and Cultural Practices

It is also important to distinguish that the benefits of the Japanese and Mediterranean dietary patterns go beyond their polyphenol concentration. Both lifestyles encourage social cohesion, physical activity, and stress decrease—all aspects that encourage healthy aging. Consequently, the adoption of polyphenol-rich dietary paradigms should be noticed as a small part of a holistic strategy to potentiate longevity. Politicians, legislators, and professionals in nutritional sciences should contribute to developing national and international guidelines that combine dietary recommendations with a salubrious style of life to optimize public health.

### 11.4. Limitations and Emerging Directions

Numerous critical research open questions remain. In particular, *(i)* large-scale, long-term controlled randomized trials with strong endpoints (i.e., age-related morbidity and mortality) are necessary to clearly indicate the causal correlation between polyphenol supplementation and potentiated life expectancy; *(ii)* the disclosure of consistent biomarkers of polyphenol efficiency, including, for example, HNE, could address early practices of intervention to shape personalized strategies; and *(iii)* since the field of nutrigenomics is in rapid progress, the investigation of putative gene–diet interactions may eventually expose new levels of information and opportunities to add other bricks in the regulation of aging by dietary polyphenols.

## 12. Discussion and Conclusions

The evidence presented in this work emphasizes the encouraging role of plant polyphenols and other plant-derived compounds such as phytosteroids (e.g., 20-Hydroxyecdysone, a natural substance found in spinach [307]) in promoting longevity. Both the traditional Japanese lifestyle and the Mediterranean diet offer convincing real-world examples of how a polyphenol-rich nutritional paradigm can regulate key biological pathways—such as inflammation, oxidative stress, and mitochondrial functionality—that play crucial roles in aging and chronic disorders. However, the exact molecular interactions continue to be an active area of research, but preclinical and clinical findings support the hypothesis that regular intake of diverse polyphenols may be a practicable approach for extending health span and enhancing life expectancy.

According to the intricacies of polyphenol bioavailability and metabolic interindividual disparities, future research should focus on integrating novel delivery systems with personalized nutrition methods. Similarly, a wider public health emphasis on whole-diet paradigms balancing bioactive chemicals with a non-stressing lifestyle and cultural practices seems necessary. These indications aim for a future in which the supplementation of plant polyphenols through a personalized dietary pattern or as optimized nutraceuticals should develop different preventive medicine milestones as a basic part of our strategy to counteract age-related disorders.

In conclusion, the convergence of findings from several disciplines, ranging from epidemiology to molecular biology, supports the hypothesis that plant polyphenols can be considered pivotal elements in the mission for longevity. It is conceivable that lifestyle-related diseases such Alzheimer’s disease, type 2 diabetes, and nonalcoholic steatohepatitis are parallel pathological phenomena induced by HNE accumulation. Therefore, the prevention of Hsp70.1 carbonylation by plant polyphenols may contribute to inhibiting the occurrence of diverse lifestyle-related conditions. The lessons emerging from Mediterranean and Japanese nutritional paradigms not only reinforce our comprehension of the aging process but could also disclose innovative interventions that might ultimately improve the duration and quality of human life.

## Figures and Tables

**Table 2:** Selected clinical trials of major polyphenols. ↑ indicates elevation. ↓ indicates reduction.

Polyphenol Type	Formulation	Dose (Daily)	Population and Intervention	Primary Outcome	References
Resveratrol	Purified capsule	150 mg	Overweight adults; several weeks	↑ SIRT1 activity, ↓ CRP	[217,218,219,220]
EGCG	Green tea extract	100/600 mg	Mild cognitive impairment; thalassemics; diabetics; 6/12 months	↑ Cognitive scores, ↓ oxidative markers	[221,222,223,224]
Hydroxytyrosol	Olive oil phenolic extract	10/15 mg	Metabolic syndrome; 8/12 weeks	↓ ox-LDL, ↓ IL-6	[225,226]
Soy Isoflavones	Soy protein isolate	60/90 mg	Post-menopausal women; 1 year	↑ Bone mineral density, ↓ LDL-cholesterol	[227,228]

## Data Availability

No new data were created or analyzed in this study. Data sharing is not applicable to this article.
